# Effects of Tailored Rehabilitation Strategies in a Conservatively Managed Traumatic Brachial Plexus Injury in a 27-Year-Old Male Patient: A Case Report

**DOI:** 10.7759/cureus.78733

**Published:** 2025-02-08

**Authors:** Anjali Rai, Raghumahanti Raghuveer, Nikita Gangwani, Pradhyum D Kolhe

**Affiliations:** 1 Department of Neurophysiotherapy, Ravi Nair Physiotherapy College, Datta Meghe Institute of Higher Education and Research, Wardha, IND

**Keywords:** brachial plexus injury, fracture, physiotherapy, pre- and postganglionic injury, rehabilitation

## Abstract

Traumatic brachial plexus injury (TBPI) is a serious neurological condition most often resulting from trauma. This condition is among the most debilitating injuries affecting the upper limb. The injury is typically categorized as preganglionic or postganglionic based on the site of trauma, proximal to or distal to the dorsal root ganglion (DRG). TBPI results in movement deficits of the upper limb with impaired muscle strength and sensitivity. This case report details the extensive rehabilitation of a 27-year-old male patient who was involved in a road traffic accident, resulting in injuries to the left upper extremity characterized by a loss of motor function and sensation. Additionally, the incident caused injuries to the left lower extremity, leading to a mid-shaft femur fracture and fractures of the metatarsals. The patient received a comprehensive clinical evaluation, along with diagnostic tests and imaging studies, which resulted in a confirmed diagnosis of pre- and post-ganglionic injury to the left upper extremity, as well as polytrauma affecting the left lower extremity. Tailored rehabilitation strategies were employed to address the diverse symptoms, including multi-sensory strategies, sensory re-education, and graded motor imagery rehabilitation. Progressive improvement of the range of motion, strength, and endurance in the lower extremities, along with the enhancement of neuromuscular control, is essential. This rehabilitation program can be used as a reference for establishing early treatment strategies.

## Introduction

Brachial plexus injury (BPI) is classified as a serious peripheral nerve injury that results in functional impairments of the upper limb and considerable disability in individuals of all ages, including both adults and children. It exerts a substantial influence, resulting in serious and enduring restrictions on arm functionality, thereby impacting daily activities and the overall quality of life for individuals affected [1]. Brachial nerve complex injuries vary in severity and etiology, spanning from mild to severe. Regrettably, traumatic cases of BP injuries are increasing, resulting in significant social and financial burdens and profoundly impacting individuals' quality of life [2]. The occurrence of BPIs has seen a considerable rise, largely attributed to the increase in high-velocity motor vehicle collisions in the 20th and 21st centuries [1]. It is estimated that motorcycle accidents, sporting incidents, or industrial accidents account for 44%-70% of all traumatic BPI (TBPIs). Specifically, 22% of motorcycle injuries result in BPI, accounting for approximately 4.2% of cases [3]. BPIs can result from various factors, including contact sports, automobile accidents, and birth complications. These injuries are broadly categorized into traumatic causes, such as car accidents and contact sports, and non-traumatic causes, such as obstetric palsy. The sensitive neural network can be damaged by stretching, compression, and laceration. Traumatic injuries can be categorized as either closed or open, based on the mechanism that caused them [3]. Closed injuries typically result from stretching or traction forces that violently pull the head and neck away from the shoulder, while open injuries are commonly the result of gunshot or stab wounds [4].

Traumatic injuries to the brachial plexus have a profound impact on the functionality of the upper limb and can result in disability. Common symptoms associated with such injuries may include restricted movement of the upper limb, as well as reduced muscle strength and sensitivity [5]. BPI is fundamentally categorized into preganglionic and postganglionic types, making the early determination of the injury level crucial for choosing the correct treatment strategy [6]. BPIs are categorized based on their distance from the dorsal root ganglion (DRG): postganglionic lesions are considered distal, while preganglionic lesions are deemed proximal. Both types, irrespective of their location, result in a loss of muscular function. Preganglionic injuries cause the nerve to be detached from the spinal cord, separating motor nerve fibers from motor cell bodies in the anterior horn cells. Lesions affecting postganglionic neurons result in the impairment of both motor and sensory nerve cells, causing disruptions in motor action potentials, as well as sensory nerve action potentials (SNAPs) [6].

Recent advances in the management of BPI have recently enhanced the outlook for functional motor recovery [7]. This encompasses conservative management, which is non-surgical, as well as non-conservative management, which involves surgical interventions. The latter includes procedures such as neurolysis, nerve repair, the application of nerve grafts, and nerve transfer, in addition to palliative surgical techniques aimed at achieving optimal functional outcomes. These may involve tendon transfer, functioning-free muscle transplantation, and arthrodesis [8]. In instances of open BPI wounds, prompt surgical intervention is essential. Conversely, for closed BPI wounds, provided that there are no other urgent injuries, immediate surgical repair may not be necessary. This approach involves assessing the condition, addressing pain management, and initiating rehabilitation. In cases where functional recovery or neurological improvement is insufficient, surgical intervention may be contemplated after a period of three to six months. This timeframe provides an opportunity to evaluate the likelihood of natural recovery and to identify the most suitable strategy for achieving the best long-term results [9]. Conservative treatment for BPI includes extensive physical and occupational rehabilitation. Commonly used techniques are joint mobilization, neurosensory motor stimulation, kinesio-taping, electrostimulation, splint immobilization, multi-sensory strategies, and graded motor imagery [10]. A collaborative approach with families is essential, which includes teaching and training them to maintain interventions at home.

Rehabilitation is essential for recovery after a BPI or trauma, with early intervention recommended to limit the risk of further complications. Therapists play an important role in helping patients return to work by making a favorable office environment and applying appropriate adjustments or orthoses to improve functional capacities [11]. A tailored rehabilitation for BPI encompasses several critical aspects: preventing muscle atrophy, managing pain, restoring somatosensory deficits, minimizing developmental disregards, and providing post-operative care.

## Case presentation

The examination was divided into sensory and motor assessments, including neurological and musculoskeletal assessments. A physical examination was conducted, and the therapist took written and verbal consent from the patient. The clinical presentation and subsequent surgical events are detailed comprehensively in Table 1 as the timeline of events. A comprehensive musculoskeletal and neurological assessment was performed. Pain assessment using the visual analog scale (VAS) rated his pain 5 out of 10 at rest and 8 out of 10 during activity. The pain was sudden in onset due to trauma, exacerbated by lower extremity movements, and alleviated by rest and medications. The pain was located at the lateral aspect of the thigh from the greater trochanter to the upper lateral aspect of the thigh and over the lateral aspect of the left dorsum of the foot and is described as dull aching. The patient also exhibits paraspinal muscle spasms. His range of motion was painful and incomplete.

**Table 1:** Timeline of events ORIF: Open reduction internal fixation, MRI: Magnetic resonance imaging, AVBRH: Acharya Vinoba Bhave Rural Hospital

Dates	Events
6/03/24	Met with a road traffic accident
6/03/24	Brought to AVBRH casualty with loss of consciousness
6/03/24	Regained consciousness and was shifted to a ward and medications were prescribed
7/03/24	X-ray of the left lower extremity was done
12/03/24	ORIF with femur interlock nailing was done
15/03/24	MRI cervical spine and MRI of the brachial plexus
13/03/24	A physiotherapy call was given

**Figure 1:**
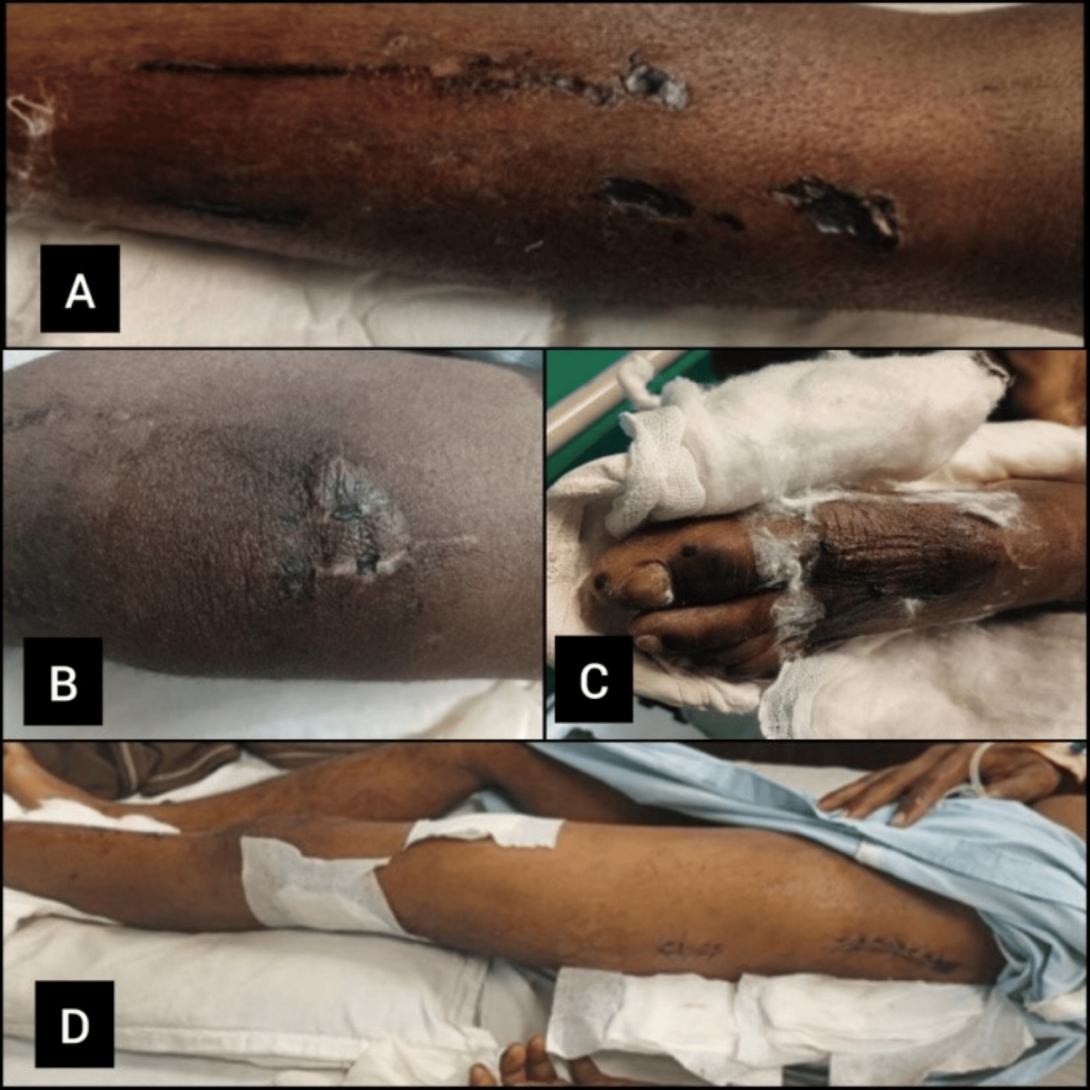
Patient wound images 1A shows abrasions over the anterior surface of a tibial shin, 1B shows abrasions over the left knee, 1C shows sutures present over the dorsal aspect of the ankle, and 1D shows the suture line present from the greater trochanter.

On observation, the patient was conscious and oriented. The patient was observed in the highest independent attainable position (i.e., sitting position), and the left shoulder was slightly lower than the right shoulder and in a protracted position. The patient was inspected in a supine position with adequate exposure. Abrasions were present over the anterior surface of a tibial shin, as shown in Figure 1A, and abrasions were present over the left knee, as shown in Figure 1B. Sutures were seen at the following locations: over the dorsal aspect of the ankle (shown in Figure 1C), and a suture line is present from the greater trochanter (shown in Figure 1D) to the upper lateral aspect of the thigh.

On palpation, tenderness grade I is present from the greater trochanter to the upper lateral aspect of the thigh, over the lower lateral aspect of the thigh, and the dorsal aspect of the ankle. Manual muscle testing (according to the Kendall muscle grading system) of the left (affected side) lower extremity: hip flexors, hip abductors, and ankle dorsiflexion were grade = 2+ (moves through a partial range of motion against gravity), whereas hip extensors, hip adductors, knee flexors, knee extensors, ankle plantar flexor, evertors, and invertors show grade = 2 (supported in the horizontal plane - movement in partial range), according to the Kendall muscle grading system. Manual muscle testing of the left (affected side) upper extremity was done - shoulder flexors and extensors only flicker were grade = 1, whereas shoulder abductors, shoulder adductors, shoulder internal and external rotators, elbow flexors, elbow extensors, wrist flexors, and extensors were grade = 0 (no contraction). Sensory examination revealed loss of superficial and deep sensations of the left upper extremity from C5 to T1 of the left affected extremity. 

Upon examination, the range of motion of the bilateral affected and unaffected upper and lower extremities is shown in Table 2. All the normal ranges are also given as references.

**Table 2:** Range of motion assessment of the bilateral upper and lower extremity NA: Not Assessable

Movement	Right	Normal	Left
Hip flexion with knee flexion	0-120°	120-140°	0-50°
Hip flexion with knee extension	0-90^0^°	90°	0-45°
Hip extension	0-25°	0-30°	0-10°
Hip abduction	0-40°	40-45°	0-15°
Hip adduction	0-25°	20-25°	0-20°
Hip internal rotation	0-40°	30-45°	NA
Hip external rotation	0-40°	35-50°	NA
Knee flexion	0-130°	0-135°	0-20°
Knee extension	135-0°	135-0°	20-0°
Ankle plantarflexion	0-50°	45-55°	0-20°
Ankle dorsiflexion	0-20°	15-20°	0-5°
Ankle inversion	0-30°	30-35°	0-15°
Ankle eversion	0-15°	15-20°	0-5°
Shoulder flexion	0-170°	0-180°	0-5 (trick movement)
Shoulder extension	0-45°	0-50°	0-5 (trick movement)
Shoulder abduction	0-170°	0-180°	0
Shoulder adduction	170-0°	180-0°	0
Shoulder internal rotation	0-80°	0-90°	0
Shoulder external rotation	0-80°	0-90°	0
Elbow flexion	0-130°	0-140°	0
Elbow extension	135-0°	140-0°	0
Wrist flexion	0-65°	0-75°	0
Wrist extension	0-65°	0-70°	0

Reflexes 

Reflexes of bilateral upper and lower extremities are shown in Table 3.

**Table 3:** Reflexes ++ means normal reflexes; NA: Not Assessable

Reflex	Nerve	Right	Left
Biceps (C5-C6)	Musculocutaneous	++	Absent
Supinator (C5-C6)	Radial	++	Absent
Triceps (C7-8)	Radial	++	Absent
Finger flexion (C7-8)	Median and ulnar nerve	++	Absent
Knee (L2-4)	Femoral	++	N/A
Abdominal (T6-T12)	Thoracic spine nerve roots	++	++
Plantar	Sacral nerve	Flexion	Flexion

Individual nerve testing of the brachial plexus

Individual nerve testing of the brachial plexus from the C5 root to the T1 root is done, and all the findings are listed in Table 4.

**Table 4:** Testing of the individual nerve of the brachial plexus

Nerve	Test	Findings
Dorsal scapular nerve (C5)	Asking the patient to retract the shoulder and/or elevate the shoulder	The patient was able to perform shoulder elevation
Long thoracic nerve (C5-C7)	Wall push-up test for serratus anterior test	The patient was unable to perform the test
Suprascapular nerve (upper trunk of BP)	Ask the patient to abduct the shoulder	The patient was unable to perform an abduction
Posterior cord nerve (upper subscapular nerve, thoracodorsal nerve, lower subscapular nerve)	Subscapularis muscle: (1) Test: Lift off test. (2) Test: Belly press test	(1) The patient was unable to lift the hand off the lower back. (2) The patient was unable to perform the test
	Latissimus muscle: Abduct the arm and then ask the patient to adduct the arm against resistance	The patient was unable to perform the adduction
	Teres major: Adducting the elevated arm against resistance	The patient was unable to perform the adduction
	Teres minor: Horn blowers test (the patient's elbow is flexed 90 degrees, and then the patient is asked to rotate the shoulder)	The patient was unable to perform shoulder rotation
Lateral pectoral nerve (lateral cord)	Ask the patient to perform shoulder flexion, adduction, and Internal rotation)	The patient was unable to perform movement
Musculocutaneous nerve (C5-C7)	Flexion and adduction of the shoulder joint, flexion and supination of the elbow joint	The patient was not able to perform movements
Axillary nerve (C6-C8)	Lateral and external rotation	The patient was not able to perform
Radial nerve (C6-C8)	Extension of elbow joint, wrist extension, finger extension	The patient was unable to perform
Median nerve (C5-T1)	Wrist and finger flexion, opposition, flexion, and abduction of the thumb	The patient was unable to perform
Ulnar nerve (C8-T1)	Froment’s sign	The patient was unable to perform

Investigations

**Figure 2:**
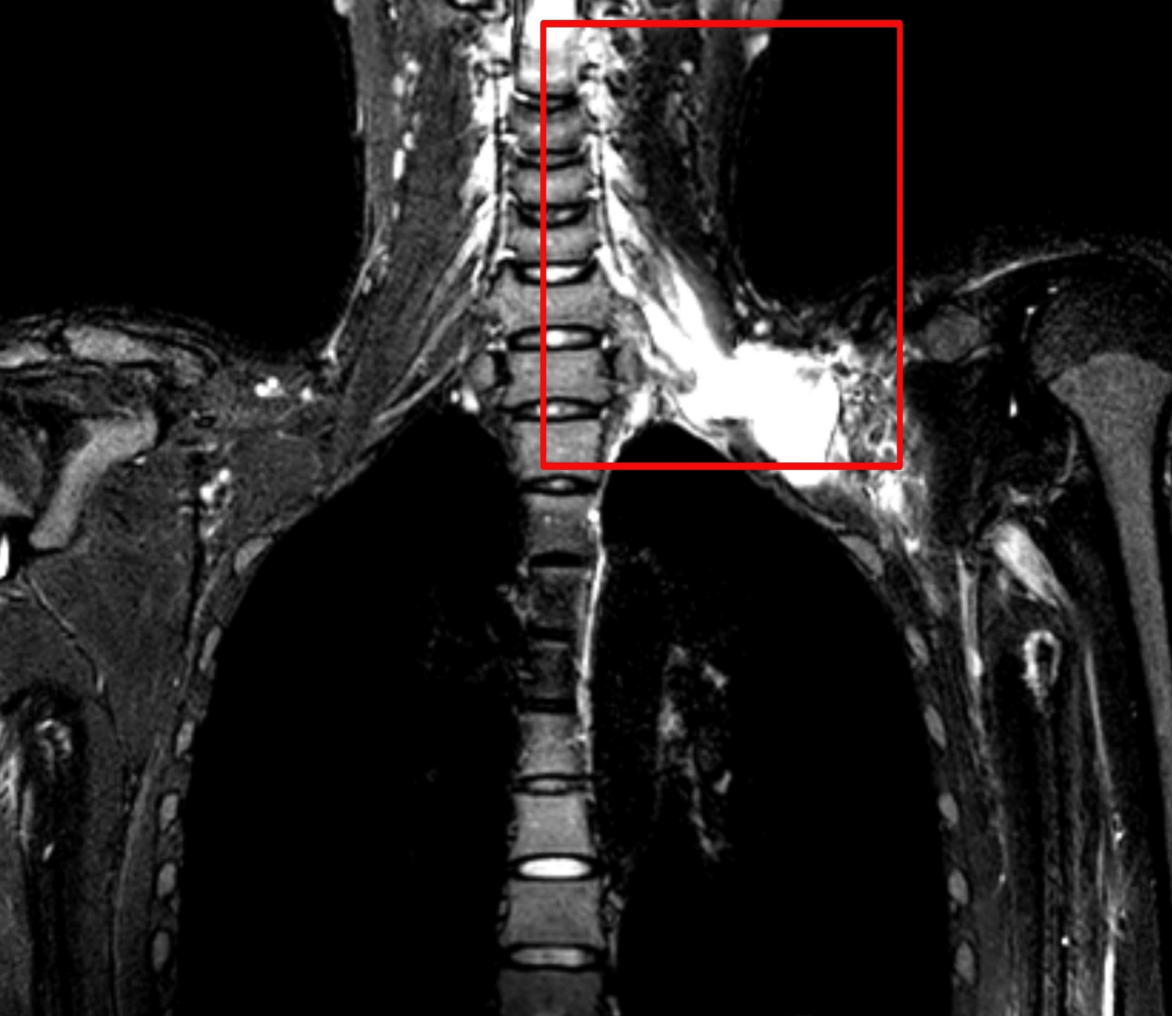
MRI of the brachial plexus Magnetic resonance imaging (MRI) shows T2 hyperintense cystic lesions noted at the roots of the left C6, C7, and C8 nerve levels, continuing along the nerve roots and reaching up to the left scalene triangle, showing signs of pseudomeningocele. Edema was noted in the left scalene triangle and in the rest of the trunk, divisions, and cords.

**Figure 3:**
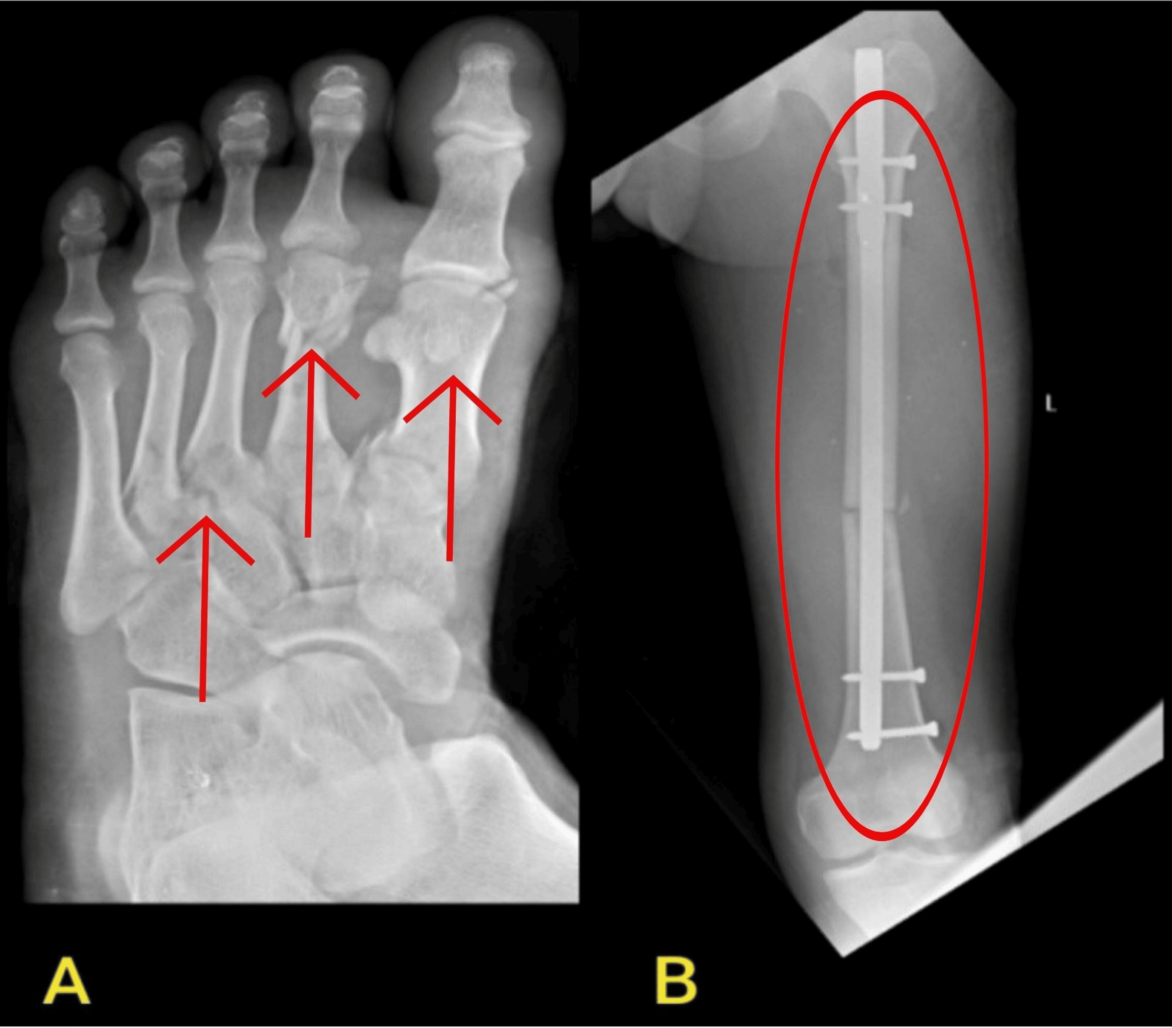
X-ray of the left foot in the AP view and the left side shaft of the femur A: Left foot in the AP view showing the 1st, 3rd, and 4th metatarsal fracture, 2nd metatarsal comminuted fracture, and 1st proximal phalanx fracture. B: Left side in the AP view showing open reduction internal fixation with femur interlock nailing for the fracture of the femoral shaft on the left side.

Magnetic resonance imaging (MRI) of the brachial plexus in Figure 2 and X-ray of the left foot in AP view and the left side shaft of femur were shown in Figure 3.

Physiotherapy management

A tailored phase-wise rehabilitation protocol was designed as per the above case scenario. Early pain management and joint stabilization prevent complications and set the stage for more intensive rehabilitation. Sensory and motor re-education starts as soon as possible to maximize the potential for recovery. Lower limb exercises are essential to restore function, prevent complications associated with immobility, and prepare eventful weight-bearing activities. Phase I (0-4 weeks) is given in Table 5.

**Table 5:** Phase I (zero to four weeks) nm: nanometer, J/mm^2^: joule per square millimeter; PNF: Proprioceptive neuromuscular facilitation

Goals	Intervention	Rationale	Dosage
To develop strategies to manage and cope with pain or discomfort	Patient and caregiver education	Educate the patient about his condition, potential complications, and preventive strategies, as well as the physiotherapy protocol to be followed	-
To prevent complications of joint contracture and subluxation	Immobilization through sling or hemi-sling/Elpeau’s bandaging	To prevent joint contracture and uncontrolled limb motion or positions, as well as minimize glenohumeral subluxation	Throughout the day
To prevent muscle de-conditioning	Active cervical retraction with the patient sitting with scapulae retracted and unilateral scapular circles	For improving stability and facilitating motor re-education and scapular synergy	10-15 times, with 5 seconds hold
To reduce inflammation and edema and to facilitate nerve regeneration	Low-intensity laser therapy over nerve roots C5, C6, C7, and T1	It reduces pain and swelling and can progressively improve nerve function	Wavelength, 780 nm power, 250 mV, 450 J/mm^2^ for 20 minutes
To regain neuromuscular control	Joint compression technique. Lateralization training. Rood’s facilitatory technique uses cotton balls (slow-light touch) and rotatory brush (fast brushing) on respective. Sensory re-education with different textures, such as sandpaper, silk, and net). Motor re-education through PNF rhythmic initiation Technique (active assisted D1-D2 flexion/extension) and range of motion exercises of bilateral upper extremity	To improve proprioception, joint stability, and muscle tone. It also enhances limb position awareness, improves motor function, and increases joint stability. Used for implicit motor imagery. This technique increases the modulation of muscle spindle sensitivity to increase muscle activity and regain motor control. For the re-establishment of impaired afferent sensory pathways or to restore sensory integration. To stimulate and improve neuromuscular control and to restore voluntary and active range of motion	10 repetitions with 30-second holds. 5-10 minutes twice daily. 3-5 times, followed by a 30-second break. 3-5 times, followed by a 30-second break. 10 repetitions of 2 sets
To reduce pain and swelling to promote healing and comfort of the lower extremities	Cryotherapy (icing with isometrics of quadriceps). Bandaging around the thigh and shin. Elevation of the affected limb	Reduces pain and swelling by constricting blood vessels, enhancing comfort, & aiding early mobilization. Bandaging supports the limb, prevents swelling, improves venous return, reduces edema, and stabilizes the limb. Elevating the affected limb twice daily and for 7-8 hours during rest promotes venous return, reduces fluid buildup, decreases swelling and pain, and improves rehabilitation effectiveness	For 10-15 minutes twice a day. For 7-8 hours (when the patient is resting). For approximately 7 hours (when the patient is resting)
To enhance the strengthening of quadriceps and hip abductors	Strengthening the quadriceps sets with holds. Hamstring sets with holds. Gluteus maximus set with holds	It strengthens and tones the quads for daily activities like walking, stairs, and standing. The hamstring strengthens back thigh muscles, aiding in knee bending, hip extension, and overall stability during various movements. The glute holds target hip extension and stability, enhancing strength for improved walking, running, and standing	10 repetitions of 2 sets with hold of 10 seconds each
To restore full-knee extension to maintain joint mobility and prevent long-term stiffness or contractures	Range-of-motion open kinetic chain exercise: Straight leg raise (in multiple angles), heel slides, prone knee bending, terminal extension in sitting	The range of motion exercises aim to improve flexibility, joint mobility, and functional movement through exercises	10 repetitions of 2 sets with hold of 10 seconds each
To maintain the flexibility of the affected extremity	Stretching the hamstring muscle gastrocnemius and soleus	The hamstring stretch improves knee and hip flexibility, while the gastrocnemius and soleus stretch enhances ankle flexibility and gait efficiency	30 seconds hold of 3 sets
To facilitate a return to normal walking patterns and promote early mobility to prevent complications associated with prolonged immobility	Gait training: Initiate with non-weight bearing with a walker, progress to partial weight bearing (25%) in 2 weeks	Gait training starts with non-weight bearing using a walker for safety, progressing to 25% weight bearing after two weeks to stimulate healing and improve strength and balance, aiding transition to normal gait	Hallway ambulation

The rehabilitation of Phase II from four weeks to eight weeks is given in Table 6. 

**Table 6:** Phase II (four to eight weeks) VMO: Vastus medialis oblique

Goals	Intervention	Rationale	Dosage
To enhance recovery of motor function: Retard muscle weakness/wasting and promote muscle re-education	Electrical stimulation, long duration 300 ms interrupted galvanic (IG) stimulation at each motor point	Used for motor relearning and to elicit contraction of targeted muscle/nerve. It also promotes nerve healing and regeneration	30 contractions at each motor point
Gradual restoration of upper extremity strength and endurance	Graded motor imagery technique includes imagination of movements without performing them and mirrors visual feedback	Used to facilitate motor control. It provides immediate feedback and facilitates re-learning through visual and auditory feedback	Each movement, 10 times twice a day
Implement strategies for sensory and motor re-education	Sensory re-education utilizing a sensory kit that corresponds to the dermatomal distribution, incorporating various textures and shapes, both with the eyes open and closed	For re-establishment of impaired afferent sensory pathways or to restore sensory integration and to accelerate sensorimotor recovery	3-5 times, followed by a 30-second break for each dermatome distribution
Restoration of functions as soon as neural regulation takes place	Rood's facilitatory approach with (fast tapping over the muscle belly)	For facilitating and re-educating muscles to gain voluntary muscular contractions	10 repetitions of 2 sets
Improving joint mobility: Restore and enhance the range of motion in the affected joints	Wall slides terminal knee extension on Swiss ball patellar mobilization and Mulligan-Bent leg raises for hamstring mobility. Standing - hip flexion, hip extension, hip abduction, hip adduction (without resistance), standing leg curls	The protocol comprises mobility-enhancing exercises. Exercises such as wall slides, terminal knee extension on a Swiss ball, patellar mobilization, Mulligan-Bent leg raise, standing hip flexion, extension, abduction, and adduction, along with standing leg curls, target specific muscle groups to improve joint flexibility, stability, and overall lower limb function, aiding in injury prevention and promoting functional movement patterns	10 repetitions of 2 sets
Improving muscular strength: Rebuild and strengthen the muscles surrounding the affected area	Strengthening the quadricep fixation hamstring curl in standing VMO strengthening. Standing - hip flexion, hip extension, hip abduction, hip adduction (with resistance). Muscle energy technique for quadriceps and the hamstring	The strengthening protocol consists of quadricep fixation for knee stability, hamstring curl in standing for lower limb strength, VMO strengthening for patellar stability, and standing hip flexion, extension, abduction, and adduction with resistance for hip muscle strength and mobility. Additionally, muscle energy technique for the quadriceps and hamstring improves muscle strength, flexibility, and neuromuscular control, contributing to rehabilitation and injury prevention	10 repetitions of 2 sets
Retaining function of physiological movement pattern while walking	Gait training: Initiate partial weight bearing 50-75% with a walker	Initiating gait training with partial weight bearing (50-75%) using a walker for 2-3 rounds in the hallway ensures stability, balance, and confidence, promoting muscle memory and functional recovery for independent ambulation	2-3 rounds in the hallway
To regain neuromuscular coordination and strength	Balance training: Single limb standing. Step-up and step down and stepping sideways	Balance training with single-limb standing, step-ups, and side steps enhances proprioception, stability, and neuromuscular control, reducing fall risk and improving functional mobility	10 repetitions of 2 sets
Promote resorption of the bone and improve endurance: Enhance the natural process of bone resorption to facilitate bone remodeling	Endurance training: Stationary bicycling - in a clockwise direction, as well as an anticlockwise direction	Stationary bicycling in both clockwise and anticlockwise directions for 15 minutes enhances cardiovascular endurance and promotes balanced muscle development	15 minutes

The rehabilitation program of Phase III (8-12 weeks) is given in Table 7.

**Table 7:** Phase III (four to eight weeks) AO–PNS: Action observation combined with peripheral electrical nerve stimulation

Goals	Intervention	Rationale	Dosage
Regain strength and neuromuscular control to facilitate motor re-education	The AO-PNS technique involves the observation of actions accompanied by peripheral nerve stimulation, showcasing a repetitive movement pattern while electrical stimulation is administered	Electrical stimulation not only promotes motor re-education but also facilitates axon growth during the process of nerve repair, thereby expediting motor recovery	10 repetitions of 2 sets
Foster a proactive approach to health and wellness, incorporating regular exercise and preventive measures, for the gradual restoration of movements	Kinesiotherapy exercises are conducted in positions that eliminate or reduce the effects of gravity, with a gradual increase in training intensity using light weights, ultimately advancing to an antigravity position	To maintain and promote range of motion, joint integrity, and strength. To rehabilitate and enhance mobility, endurance, and strength	10 repetitions of 2 sets
Promote strengthening	Isometrics contractions of shoulder flexion, external/internal rotations, adduction and abduction	To enhance muscle activation, as well as promote strengthening	5 repetitions each with 30 seconds of hold
Restoration of muscular strength: Rebuild and enhance muscle strength to support overall function and prevent re-injury	Strengthening exercises: Lower limb heel raises with holds toe raises with holds forward and backward lunges (initially without weights, progresses to with weights), squats (initially without weights, progresses to with weights), clamshell abduction/adduction with a resistance band	Lower limb strengthening exercises enhance stability and strength for weight-bearing activities, while core and pelvic exercises improve overall stability, posture, and functional movement, aiding injury prevention and daily performance	10 repetitions of 2 sets
Optimization of core and pelvic stability: Strengthen the core and pelvic muscles to provide a stable foundation for all movements and reduce the risk of injury	Core and pelvic strengthening: Glute bridge with holds, glute bridge with abduction/adduction core strengthening with isometric holds	The core and pelvic strengthening exercises aim to improve overall stability, posture, and functional movement patterns, aiding in injury prevention and promoting optimal performance in daily activities	10 repetitions of 2 sets
Optimization of coordinated movement patterns along the kinematic chain during movement	Balance training: (1) Static balance: Single-leg stands, tandem stance, beginning with stable surfaces and progressing to unstable surfaces (balance boards, foam pads). (2) Dynamic balance: Single-leg stands with reach and step-ups on unstable surfaces, progressing to dynamic movements like hopping or step-ups on unstable surfaces	Balance training progresses from static exercises like single-leg stands to dynamic movements on unstable surfaces, enhancing stability, coordination, and strength for injury prevention and better daily and sports performance	10-20 seconds hold, 2-3 sets, 2-3 times per week
Developing ergonomic postures, gait, and movements in everyday routine: To promote safe and efficient postures and movements in daily activities to prevent strain and injury	Gait training: Initiate full weight bearing without a walker	Initiating full weight bearing without a walker allows for gradual adaptation to weight bearing, promoting confidence and independence in ambulation	2 rounds initially, increase according to patient’s convenience

Outcome measure

The outcome measures throughout the rehabilitation, which was measured as day one assessment, after one-month assessment, and after two-month assessment were given in Table 8.

**Table 8:** Outcome measures throughout the rehabilitation VAS: Visual analog scale, ROM: Range of motion, MMT: Manual muscle testing, BPOM: Brachial plexus outcome measure, UEFS: Upper extremity functional scale

Outcomes	Day 1 assessment	After 1month	After 2months
VAS	On rest: 5/10	On rest: 4/10	On rest: 1/10
	On activity: 8/10	On activity: 6/10	On activity: 2/10
ROM (lower extremity)			
Hip flexion	0-50°	0-80°	0-110°
Hip extension	0-10°	0-20°	0-30°
Hip abduction	0-15°	0-30°	0-40°
Hip adduction	15-0°	30-0°	0-40°
Knee flexion	0-20°	0-60°	0-100°
Knee extension	20-0°	60-0°	100-0°
Ankle plantarflexion	0-20°	0-30°	0-40°
Ankle dorsiflexion	0-5°	0-15°	0-20°
MMT (affected side)			
Lower extremity	2/5	3+/5	4/5
MMT (affected side)			
Shoulder flexors	1/5	1+/5	1+/5
Shoulder extensors	0/5	1/5	1+/5
Shoulder abductors	0/5	1/5	1+/5
Shoulder adductors	0/5	1/5	1+/5
Barthel index	30/100	50/100	80/100
Lower extremity functional scale	10/100	40/00	60/100
BPOM	13	16	20
UEFS	4	12	24

## Discussion

BPIs are one of the uncommon conditions that are often classified as obstetric or traumatic injuries. Motorcycle accidents frequently rank among the leading causes of traumatic BPIs, particularly in adult populations. This injury to the plexus causes issues with atrophy of associated muscle groups, neurologic degeneration, and lack of sensory and motor dysfunction [12]. BPIs are challenging and involve extensive, long-term comprehensive rehabilitation. Patients often have trouble with daily activities, including returning to work, particularly when the occupation requires manual labor [13]. The primary factor contributing to traumatic BPIs resulting from road traffic accidents is the occurrence of closed injuries. Additionally, Jain et al. noted that the dominant hand is often more frequently impacted [14]. The functional outcomes of the injury are influenced by various factors, including the severity, extent, and type of nerve damage. Consequently, different patterns of injury may result in a range of neurological disorders [2]. Rich et al. mentioned that road traffic accidents can result in traumatic BPIs, which are frequently a component of a complex polytrauma presentation, including fractures and dislocations of either the upper or lower extremity [13]. The study of Solomen et al. mentioned that the mechanism of injury was forced lateral neck flexion and head traction simultaneously, which was managed conservatively. The physiotherapy rehabilitation started late in this case and caused some sort of complications, which also delayed the prognosis of the patient [15]. Additionally, Rich et al. documented a case involving a 28-year-old patient who experienced polytrauma, resulting in multiple injuries to both the upper and lower extremities. They concluded that, due to the intricacy of the polytrauma, the rehabilitation would indicate sooner. The postponement of rehabilitation may potentially exacerbate post-traumatic nerve inflammation and impede the rate of nerve regeneration [13].

In the case report presented, the forces of traction, compression and direct impact applied to the patient's neck and shoulder during the injury may have played a role in the formation of both pre- and postganglionic BPIs. As the patient was managed conservatively, we set an early comprehensive rehabilitation approach firstly to minimize the possible secondary complications such as muscle atrophy and joint stiffness. Joint stiffness is reduced, and it also accelerates the rate of nerve regeneration, leading to a better prognosis for the patient's motor and sensory recovery. To develop a plan of care, problems were identified, goals were set, and intervention was planned as needed to minimize secondary complications and for a better prognosis regarding functional motor and sensory recovery. A comprehensive rehabilitation approach focused on pain management, strategies for sensory and motor re-education, the gradual restoration of lower extremity strength and endurance, and the development of neuromuscular control. As the patient is managed conservatively, immobilization is given through sling or hemi-sling/Velpeau’s bandaging. In a scoping review, de Santana Chagas et al. mentioned that scapular muscle strengthening should be considered to gain function in the upper limb as, due to injury, it can be biomechanically imbalanced and start causing complications. Scapular strengthening for improving stability and facilitating motor re-education and scapular synergy is presented. Additionally, electrotherapeutic modalities such as low-level laser therapy and electrical stimulation are found to be effective when given on individual motor points. Laser therapy helps in reducing pain due to its anti-inflammatory actions, which result in micro-stimulation. Graded motor imagery and biofeedback strategies were used to stimulate and improve neuromuscular control and to restore voluntary and active range of motion. Sensory re-education techniques using different textures, such as sandpaper, silk, and net, are employed to re-establish impaired afferent sensory pathways and restore sensory integration.

## Conclusions

This case report underscores the critical need for a thorough and prompt rehabilitation strategy for patients suffering from TBPIs, especially those undergoing conservative treatment. By implementing customized rehabilitation techniques that emphasized pain alleviation, sensory and motor re-education, and the progressive enhancement of strength and neuromuscular coordination, notable advancements in both functional motor and sensory recovery were achieved. The application of diverse rehabilitation methods, such as electrotherapeutic modalities, graded motor imagery, and sensory re-education was vital in facilitating the patient's recovery. This rehabilitation program serves as a guideline for formulating early goal-oriented treatment strategies for patients with conservatively managed BPIs, highlighting the possibility of enhanced outcomes through a systematic and multidisciplinary methodology.
